# Association between live microbe intake and depression in COPD aged 40 years and older: evidence from NHANES 2005–2018

**DOI:** 10.3389/fnut.2025.1669775

**Published:** 2025-12-03

**Authors:** Wei Gao, Xin Ling, Yan Wang, Xiaoxia Zhao, Yushan Shi

**Affiliations:** 1 Department of Laboratory Medicine, Affiliated Hospital of Shandong University of Traditional Chinese Medicine, Jinan, Shandong, China; 2 Department of Respiratory and Critical Care Medicine, Affiliated Hospital of Shandong University of Traditional Chinese Medicine, Jinan, Shandong, China; 3 Department of Emergency and Critical Care Medicine, Affiliated Hospital of Shandong University of Traditional Chinese Medicine, Jinan, Shandong, China; 4 First Clinical Medical College, Shandong University of Traditional Chinese Medicine, Jinan, Shandong, China

**Keywords:** chronic obstructive pulmonary disease (COPD), live microbes, depression, dietary intake, NHANES

## Abstract

**Objective:**

This study aimed to investigate the association between dietary intake of live microbes and the prevalence of depression among patients with Chronic Obstructive Pulmonary Disease (COPD) aged 40 years and older, using data from the National Health and Nutrition Examination Survey (NHANES) 2005–2018.

**Methods:**

The study included 1,494 participants (representing 9.04 million COPD adults in the U.S.) aged 40 and above. Dietary intake of live microbes was categorized into low, moderate, and high groups based on 24-h dietary recall data. Depression was assessed using the Patient Health Questionnaire-9 (PHQ-9) with a score ≥10 indicating depression. Weighted logistic regression analysis was performed to evaluate the association between live microbe intake and depression, adjusting for various covariates. Additionally, subgroup analysis and sensitivity analysis were conducted.

**Results:**

Higher dietary intake of live microbes was associated with a lower prevalence of depression in COPD participants. In the fully adjusted model, compared to the low intake group, the odds ratio (OR) for depression was 0.55 (95% CI: 0.33–0.92) for the high intake group. This inverse association was still statistically significant in individuals under 65 years, males, non-obese individuals, and those without cardiovascular disease. In addition, the results of the sensitivity analysis also indicate relationship stability.

**Conclusion:**

This study indicates an inverse association between higher dietary intake of live microbes and the prevalence of depression among COPD patients aged 40 and above in the United States. Future prospective studies are needed to verify this association and explore underlying mechanisms.

## 1 Introduction

Chronic Obstructive Pulmonary Disease (COPD) is a progressive chronic respiratory disease characterized by abnormal airways and/or alveoli, leading to persistent airflow limitation (1). COPD was one of the leading causes of mortality worldwide (2). It is also a major cause of morbidity and healthcare utilization worldwide (3, 4). Compared to COPD patients without comorbidities, those with concurrent conditions are more likely to experience frequent hospitalizations and may face premature mortality (5, 6). Of such comorbidities, depression contributes to a substantial burden of COPD-related morbidity (7). Previous studies have indicated that depression may not only increase the risk of developing COPD, but is also associated with a higher likelihood of 30-day readmission and an elevated risk of acute exacerbations of COPD (8, 9). Therefore, early identification or intervention of the influencing factors of depression in COPD patients is even more crucial for early detection and treatment.

Studies have shown that consuming live microorganisms is beneficial to human health (10). This is because they can enhance intestinal function and reduce disease risk by interacting with the microbiota residing in the gut (11). Research shows that probiotics containing live microorganisms and fermented foods can alleviate symptoms of depression (12, 13). Changes in gut microbiota have also been confirmed to be associated with disease progression and related complications in COPD patients (14). Recently, Marco et al. proposed a classification system using the NHANES public database to define and estimate dietary intake of live microbes (15). Based on this classification system, multiple studies have explored the correlation between dietary live microorganisms and various common clinical diseases (16–19). Emerging evidence suggests that the gut–lung axis may play a role in modulating systemic inflammation and mental health in COPD patients, providing a biological rationale for investigating dietary live microbes in this population (20). existing studies have found that a high content of live dietary microbes significantly reduces the risk of depressive symptoms in patients with chronic airway inflammatory diseases (18).

To our knowledge, no prior study has examined whether dietary live microbe intake is associated with depression among COPD patients. This study aims to fill that gap. Which may provide new insights and approaches for the prevention and treatment of depression in this population.

## 2 Materials and methods

### 2.1 Data and study participants

NHANES is a national survey conducted biennially since 1999 in the United States, using complex multi-stage probability sampling methods to collect information about the health and nutrition status of the U.S. population. It uses a hierarchical multi-stage probabilistic design to screen participants, including oversampling of specific age and racial groups. This method allows for sample-weighted inference and provides a representative representation of the U.S. population. The Institutional Review Board of the National Center for Health Statistics (NCHS) has approved NHANES. The consent form was signed by each participant of the survey. Our research strictly adheres to the STROBE guidelines.

**Figure 1:**
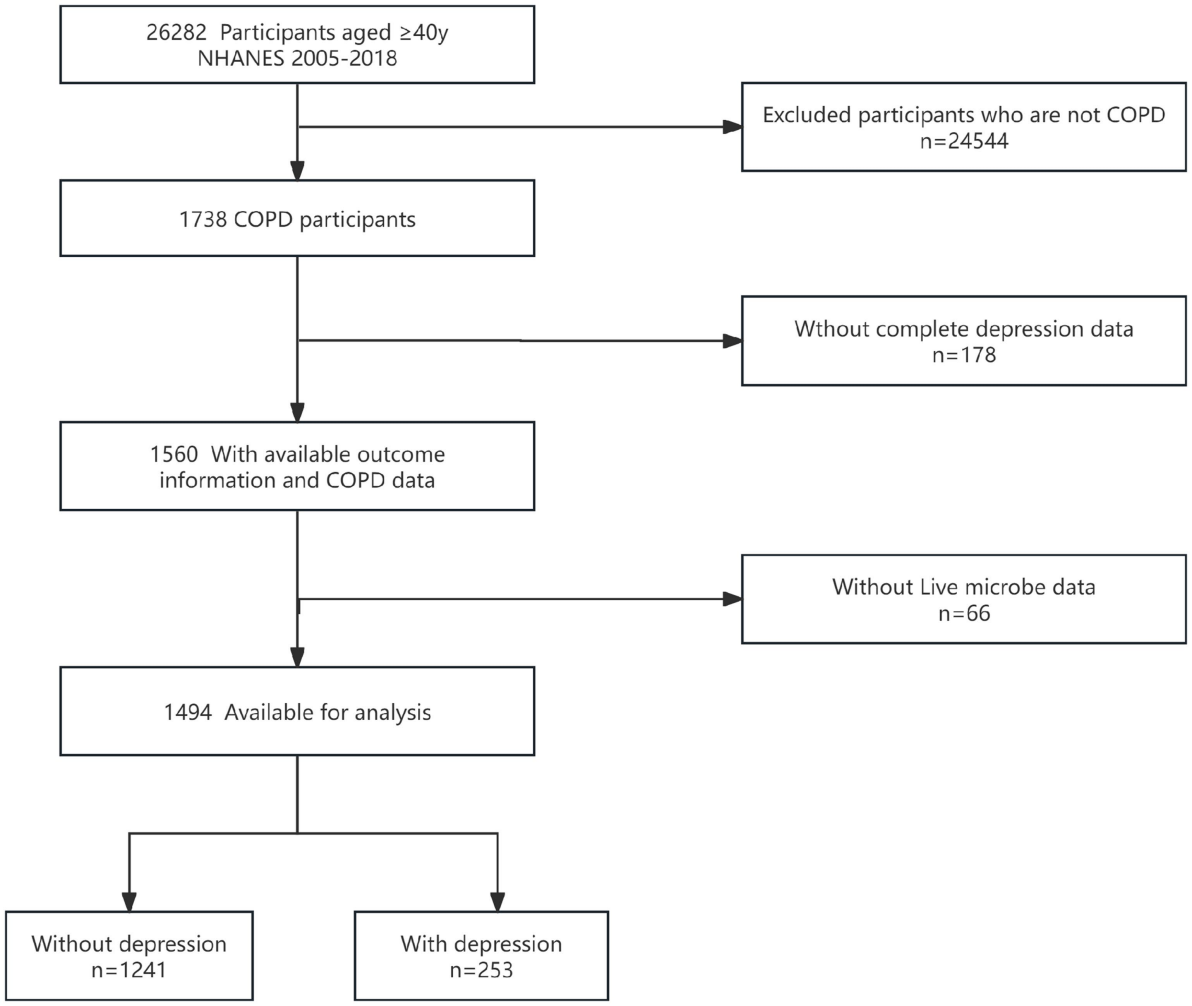
Flow diagram of the screening and enrollment of study participants. COPD, chronic obstructive pulmonary disease; NHANES, National Health and Nutrition Examination Survey.

Our study included data from seven survey cycles between 2005 and 2018. There were 26,282 participants aged 40 and above. Among them, 1,738 had COPD, 178 lacked depression information, and 66 lacked information on probiotic intake. Given that the proportion of missing covariates was less than 0.5% (*n* = 18), this study did not apply special handling for the missing data. As shown in Figure 1, after screening, a total of 1,494 participants were ultimately included in the study, there were 253 depressed individuals and 1,241 non-depressed individuals.

### 2.2 Dietary live microbe intake

Estimate dietary intake of live microorganisms using the 24-h dietary recall data from NHANES. The classification of live microorganism content in foods is derived from the study by Marco et al. (15). The estimated number of live microorganisms expressed as colony-forming units per gram (CFU/g) in 9,388 foods across 48 subgroups in the NHANES database was determined by a team of four experts (MLM, MES, RH, and CH). Experts classify food into low (Lo; < 10^4^ CFUs/g), medium (Med; 10^4^–10^7^ CFUs/g), and high (Hi; > 10^7^ CFUs/g) categories based on their live microorganism content. Generally, the lower tier mainly consists of pasteurized foods, the middle tier primarily includes fresh fruits and vegetables with their skins intact, while the higher tier comprises unpasteurized fermented foods and probiotic supplements (21). If there are uncertain or conflicting data, external consultations were conducted (22). A single 24-h recall may not reflect habitual intake and is subject to recall bias. CFU/g estimates may vary due to processing, storage, or cooking, leading to potential misclassification. In view of existing literature methods, in the study, participants were divided into three groups based on their diet live bacteria content: diet live bacteria group (all foods low), moderate dietary live bacteria group (moderate content but no high-content foods), and high dietary live bacteria group (containing high-content foods) (23).

### 2.3 Diagnosis of COPD

Based on previous literature (5–7), Only a portion of participants in NHANES have pulmonary function data, so a composite definition is used to improve diagnostic coverage. COPD is defined as having one of the following conditions: (1) Post-bronchodilator FEV1/FVC < 0.7; (2) Self-reported emphysema; (3) Age > 40 years, history of chronic bronchitis or smoking, treated with COPD medications (including mast cell stabilizers, leukotriene modifiers, inhaled corticosteroids, selective phosphodiesterase 4 inhibitors).

### 2.4 Definition of depression

Current depressive symptoms are measured using the Patient Health Questionnaire-9 (PHQ-9) (5–7). The PHQ-9 consists of nine items, each assessed on a four-point Likert scale ranging from 0 (not at all) to 3 (nearly every day). The total score of the PHQ-9 ranges from 0 to 27, with higher scores indicating greater severity. A PHQ-9 score of ≥10 was recommended as the binary threshold to define the presence of depression (24).

### 2.5 Covariates

Based on previous literature and clinical significance (25–27), this study included the following covariates: age, gender, race (Mexican American, non-Hispanic Black, non-Hispanic White, other Hispanic, other race—including multiracial), marital status (living alone or married/cohabiting), educational attainment (less than high school, high school, college or higher), poverty income ratio (PIR), body mass index (BMI), smoking, alcohol consumption, physical activity time, prior medical history, and dietary energy/protein/fat intake. The calculation method for BMI is weight (kg) divided by height (m) squared, then categorized as ≤25, >25–30, and >30 (28). PIR classification is as follows: low income (PIR ≤ 1.3), middle income (1.3 < PIR ≤ 3.5), and high income (PIR > 3.5) (29). Smoking status is divided into never smokers, former smokers, and current smokers (30). Alcohol use is categorized based on current drinking status and extent (never, former, current drinker) (31). Physical activity time was assessed by reporting the duration of weekly walking or cycling, household chores, work, and recreational activities (32). Participants were asked: “Has a doctor or other health professional ever told you that you had congestive heart failure/coronary heart disease/angina/heart attack/stroke?” If an individual answered “yes” to any of these questions, they were considered to have cardiovascular disease (CVD) (33). Diabetes is defined as the use of insulin or oral hypoglycemic agents, self-reported and physician-diagnosed diabetes, fasting blood glucose levels exceeding 7.0 mmol/L, 2-h postprandial blood glucose levels during an oral glucose tolerance test exceeding 11.1 mmol/L, random blood glucose levels exceeding 11.1 mmol/L, or glycated hemoglobin (HbA1c) levels exceeding 6.5% (34). Hypertension is defined as self-reported hypertension, or an average systolic blood pressure greater than 140 mmHg and/or an average diastolic blood pressure greater than 90 mmHg, or the use of antihypertensive medication (35).

### 2.6 Statistical analyses

All analyses accounted for the complex sampling design of NHANES. Given that live microbial intake was calculated based on dietary data, this study utilized the day 1 dietary weights. Continuous variables with normal distributions were expressed as weighted means (standard deviations), while those with non-normal distributions were presented as medians (interquartile ranges), with statistical differences described using analysis of variance (ANOVA) or Kruskal–Wallis tests. Categorical variables were reported as weighted frequencies and percentages, with differences compared using chi-square tests.

This study employed weighted logistic regression analysis to assess the association between live microbial intake and depression. Four models were constructed: the crude model was unadjusted for any variables; Model 1 adjusted for age, sex, and race; Model 2 further adjusted for education level, marital status, PIR (poverty income ratio), smoking status, alcohol consumption, physical activity, and BMI; Model 3 additionally adjusted for CVD, hypertension, diabetes, total dietary energy intake, total protein, and total fat. Trend tests were employed to assess the linear relationship between the categorical variables representing dietary live microbial intake levels and depression.

Stratified regression analyses were conducted based on age, sex, BMI, and CVD, along with interaction tests to examine heterogeneity across different subgroups.

To verify the stability of the results, we excluded participants with extremely high energy intake, those consuming less than 500 kcal or more than 5,000 kcal per day, for sensitivity analysis. Given that less than 0.5% of covariates were missing, it had little impact on the core results, so we performed complete-case analysis without imputation. However, to further verify the impact of missing covariates data on the results, a sensitivity analysis was conducted after deleting the missing cases. All analysis code is available upon reasonable request.

All statistical analyses were performed using R software (version 4.2.2) and Free Statistics software version (1.9.2). A two-sided *p*-value less than 0.05 is used as the criterion for statistical significance.

## 3 Results

### 3.1 Participant characteristics

The final sample for analysis included 1,494 individuals, representing an estimated 9.04 million COPD adults in the United States. Table 1 shows the basic characteristics of the participants based on their levels of dietary probiotic intake. The prevalence of depression was 18.6, 12.4, and 7.6% across low, medium, and high live microbe intake groups, respectively. The mean age was 61.7 years (SD = 11.1). compared with the low group, subjects in the medium-high group were predominantly male, Mexican American, married, had higher income and education levels, currently consumed alcohol, and had greater total energy, total protein, and total fat intake (all *p* < 0.05).

**Table 1:** Characteristics of participants enrolled in study according to the different dietary live microbes.

Characteristics	Total	Low live microbes	Medium live microbes	High live microbes	*p*
	(*n* = 1,494)	(*n* = 634)	(*n* = 570)	(*n* = 290)	
Weighted population, *n* (in millions)	9.04	3.49	3.34	2.21	
Age, year	61.72 (11.07)	60.68 (11.42)	62.30 (10.88)	62.49(10.69)	0.10
Sex, *n* (in millions), %					0.02
Female	4.57 (50.62)	1.98 (56.76)	1.58 (47.28)	1.02 (45.98)	
Male	4.46 (49.38)	1.51 (43.24)	1.76 (52.72)	1.19 (54.02)	
Race, *n* (in millions), %					<0.05
Mexican American	7.40 (81.88)	2.68 (76.81)	2.79 (83.72)	1.92 (87.10)	
Non-Hispanic Black	0.631 (6.94)	0.34 (9.79)	0.21 (6.21)	0.08 (3.54)	
Non-Hispanic White	0.19 (2.06)	0. 08 (2.34)	0.09(2.64)	0.02 (0.74)	
Other Hispanic	0.21 (2.37)	0. 09 (2.52)	0.07 (2.14)	0.06 (2.49)	
Other race	0.61 (6.75)	0.30 (8.53)	0.18 (5.30)	0.14 (6.13)	
Marital status, *n* (in millions), %					0.04
Married/living with partner	5.71 (63.20)	2.01 (57.55)	2.24 (67.24)	1.46 (66.02)	
Never married/other	3.32 (36.80)	1.48 (42.45)	1.09 (32.76)	0.75 (33.98)	
PIR	2.830 (1.65)	2.414 (1.58)	2.941 (1.68)	3.331 (1.54)	<0.001
PIR, *n* (in millions), %					<0.001
≤1.3	2.16 (25.72)	1.14 (34.95)	0.73 (23.72)	0.28 (13.89)	
1.3–3.5	3.06 (36.56)	1.19 (36.48)	1.12 (36.47)	0.75 (36.85)	
>3.5	3.16 (37.72)	0.93 (28.57)	1.23 (39.82)	1.00 (49.26)	
Education level, *n* (in millions), %					<0.001
Less than high school	1.83 (20.26)	0.97 (27.75)	0.65 (19.37)	0.22 (9.78)	
High school or equivalent	2.33 (25.78)	0.89 (25.62)	0.96 (28.85)	0.47 (21.40)	
Above high school	4.88 (53.96)	1.63 (46.63)	1.73 (51.78)	1.52 (68.82)	
Smoke, *n* (in millions), %					0.053
Never	1.57 (17.39)	0.55 (15.77)	0.612 (18.52)	0.40 (18.25)	
Former	4.31 (47.65)	1.49 (42.61)	1.64 (49.03)	1.18 (53.51)	
Now	3.16 (34.96)	1.45 (41.62)	1.08 (32.45)	0.62 (28.24)	
Drinking status, *n* (in millions), %					0.045
Never	0.53 (5.89)	0.26 (7.52)	0.22 (6.46)	0.05 (2.47)	
Former	2.60 (28.72)	1.04 (29.80)	1.04 (31.01)	0.52 (23.57)	
Now	5.91 (65.39)	2.19 (62.68)	2.09 (62.54)	1.63 (73.96)	
Physical activity, minutes/week	150 (0.00,600)	120 (0.00, 600)	210 (0.00, 549)	179 (0.00, 600)	0.29
BMI, kg/m^2^					0.68
<25 (normal weight)	2.29 (25.55)	0.82 (23.91)	0.92 (27.81)	0.54 (24.70)	
25–29.9 (overweight)	3.03 (33.78)	1.13 (32.70)	1.10 (33.06)	0.81 (36.57)	
*≥*30 (obese)	3.65 (40.67)	1.50 (43.39)	1.30 (39.12)	0.85 (38.73)	
Energy intake (kcal)	1855 (1,428,2,472)	1818 (1,317,2,416)	1797 (1,391, 2,366)	2036 (1,565, 2,734)	0.002
Protein intake (g/day)	69.86 (48.08, 94.32)	65.46 (43.54, 90.47)	66.11 (47.39, 88.57)	80.01 (56.49, 109.45)	<0.001
Fat intake (g/day)	71.18 (49.49,100.07)	67.17 (44.68, 95.94)	70.014 (50.30, 96.09)	78.344 (54.69, 112.62)	0.003
CVD, *n* (in millions), %					0.47
No	6.63 (73.34)	2.48 (71.01)	2.49 (74.73)	1.65 (74.91)	
Yes	2.41 (26.66)	1.01 (28.99)	0.84 (25.27)	0.55 (25.09)	
Hypertension, *n* (in millions), %					0.83
No	3.76 (41.66)	1.48 (42.53)	1.40 (41.96)	0.88 (39.84)	
Yes	5.27 (58.34)	2.00 (57.47)	1.94 (58.04)	1.33 (60.16)	
DM, *n* (in millions), %					0.36
No	6.70 (74.11)	2.53 (72.62)	2.43 (72.82)	1.73(78.42)	
Yes	2.34 (25.89)	0.96 (27.38)	0.91 (27.18)	0.48 (21.58)	
Depression					0.001
No	7.80 (86.40)	2.84 (81.40)	2.92 (87.60)	2.04 (92.40)	
Yes	1.23 (13.60)	0.65 (18.60)	0.41 (12.40)	0.17 (7.60)	

Normally distributed continuous variables are described as mean ± SD, and continuous variables without a normal distribution are described as median (interquartile range). Categorical variables are presented as number in millions (%).

PIR, Poverty Income Ratio; BMI, body mass index; CVD, cardiovascular disease, DM, diabetes mellitus.

### 3.2 Association between the intake of live microbe and depression

The results of the weighted logistic regression analysis are shown in Table 2. Overall, higher intake of dietary live microbe was associated with lower prevalence of depression in all models. In the unadjusted model, compared to the low dietary live microbe group, the OR (95% CI) for depression was 0.62 (0.40–0.96) for the medium dietary live microbe group and 0.36 (0.23–0.58) for the high dietary live microbe group. In the fully adjusted model, compared to the low dietary live microbe group, the OR (95% CI) for depression was 0.83 (0.56–1.24) for the medium dietary live microbe group and 0.55 (0.33–0.92) for the high dietary live microbe group. A significant linear trend was observed across intake groups (*p* for trend < 0.05).

**Table 2:** Association between dietary live microbe intake and depression prevalence in COPD participates.

Variables	Crude model		Model 1		Model 2		Model 3	
	OR (95%CI)	*p*	OR (95%CI)	*p*	OR (95%CI)	*p*	OR (95%CI)	*p*
Live microbes groups								
Low	Reference		Reference		Reference		Reference	
Medium	0.62(0.40, 0.96)	0.034	0.63(0.40, 0.99)	0.046	0.80(0.53, 1.21)	0.296	0.83(0.56, 1.24)	0.36
High	0.36(0.23, 0.58)	<0.001	0.37(0.23, 0.58)	<0.001	0.55(0.32, 0.93)	0.028	0.55(0.33, 0.92)	0.022
*P* for trend		<0.001		<0.001		0.018		0.013

*Crude model:* Not adjusted.

*Model 1*: Adjusted for age, sex, race.

*Model 2*: Adjusted for age, sex, race, marital status, education level, smoking status, drinking status, physical activity time, PIR, BMI.

*Model 3*: Adjusted for age, race, marital status, education level, smoking status, drinking status, physical activity time (minutes), PIR, BMI, CVD, Hypertension, DM, total energy, total protein and total fat.

COPD, chronic obstructive pulmonary disease; OR, odd ratio; CI, confidence interval; PIR, poverty income ratio; BMI, body mass index; CVD, cardiovascular disease; DM, diabetes mellitus.

### 3.3 Subgroup analysis and sensitivity analysis

**Figure 2:**
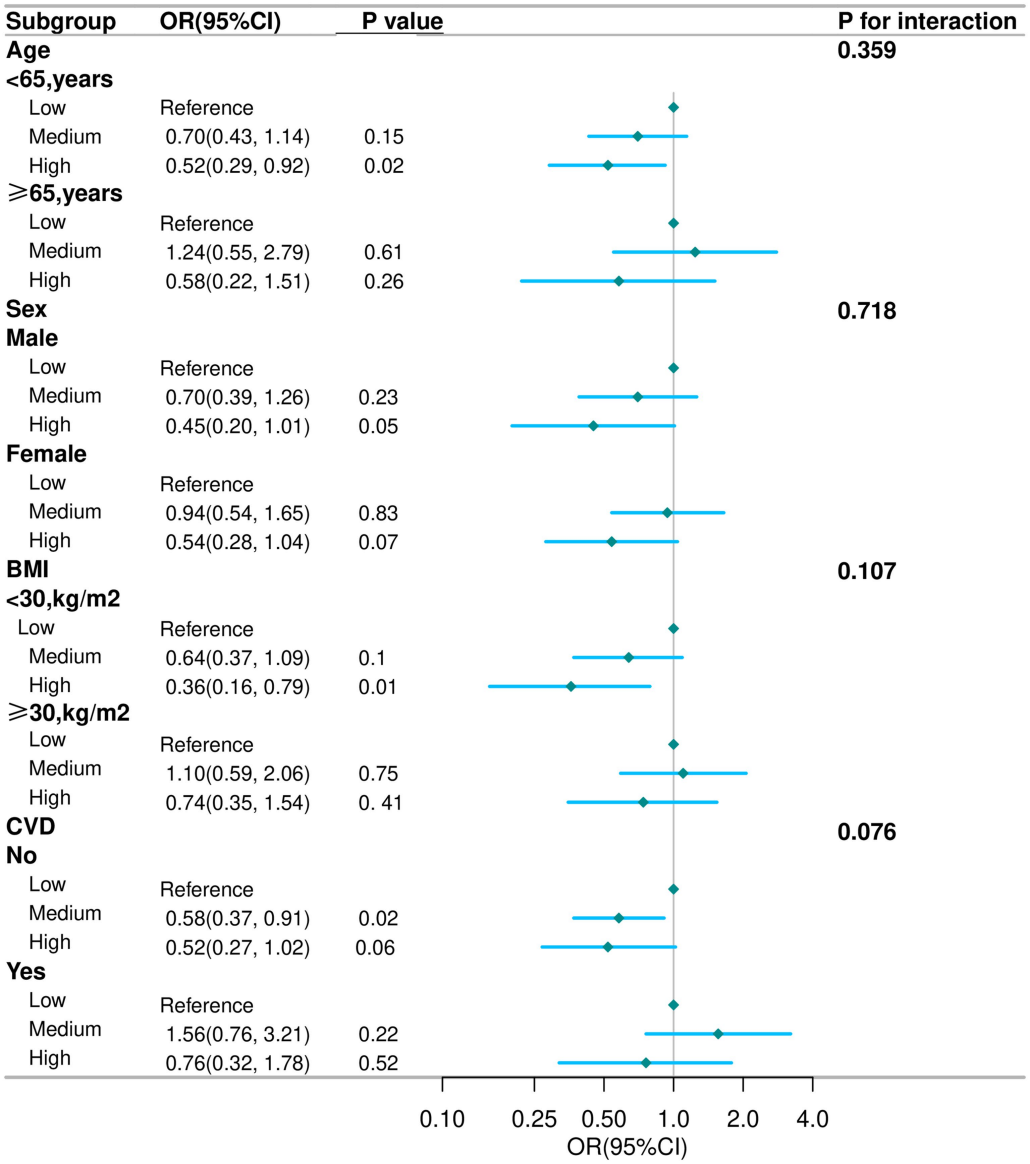
Subgroup analysis for the association between live microbe intake and the prevalence of depression in COPD aged 40 years and older. Except for stratification component itself, each stratification factor was adjusted for age, race, marital status, education level, smoking status, drinking status, physical activity time, PIR, BMI, CVD, Hypertension, DM, total energy, total protein and total fat. COPD, chronic obstructive pulmonary disease; OR, odd ratio; CI, confidence interval; PIR, poverty income ratio; BMI, body mass index; CVD, cardiovascular disease; DM, diabetes mellitus.

Subgroup analyses were conducted in several subgroups by gender (male or female), age (<65 or ≥65 years), BMI (<30 or ≥30 kg/m^2^), and CVD (No or Yes). In subgroup analyses, the inverse association between high live microbe intake and depression was generally consistent, though no significant interactions were observed (all P for interaction > 0.05) (Figure 2). Specifically, the association between live microbes and depression remains stable across different subgroups and retains statistical significance among participants under 65 years old, males, those with a BMI less than 30 kg/m^2^, and non-CVD individuals. Among participants aged <65 years, the odds ratio (OR) for depression in the high intake group was 0.52 (95% CI: 0.29–0.92); in males, the OR was 0.45 (95% CI: 0.20–1.01); among non-obese individuals (BMI < 30 kg/m^2^), the OR was 0.36 (95% CI: 0.16–0.79); in those without CVD, the OR was 0.52 (95% CI: 0.27–1.02).

To verify the robustness of the results, weighted regression analyses were conducted between dietary live microbial intake and depression after excluding participants with missing covariates (*n* = 18) and those with extreme energy intake (<500 kcal or >5,000 kcal per day, *n* = 37), respectively. As shown in Table 3, after excluding participants with missing covariates, 1,476 individuals remained. In the fully adjusted model, compared to the low dietary live microbe group, the high intake group had a 45% reduced risk of depression (OR: 0.55; 95% CI: 0.33–0.92). After excluding participants with extreme energy intake, 1,457 individuals remained. In the fully adjusted model, compared to the low dietary live microorganism group, the high intake group had a 44% reduced risk of depression (OR: 0.56; 95% CI: 0.33–0.94).

**Table 3:** Sensitive analysis of the association between dietary live microbe intake and depression.

Variables	Crude model		Model 1		Model 2		Model 3	
	OR (95%CI)	*p*	OR (95%CI)	*p*	OR (95%CI)	*p*	OR (95%CI)	*p*
Live microbes groups (*n* = 1,476)								
Low	Reference		Reference		Reference		Reference	
Medium	0.61(0.39, 0.96)	0.034	0.63(0.40, 0.99)	0.047	0.80(0.53, 1.21)	0.296	0.83(0.56, 1.24)	0.360
High	0.35(0.22, 0.55)	<0.001	0.36(0.22, 0.57)	<0.001	0.55(0.32, 0.93)	0.028	0.55(0.33, 0.92)	0.022
*P* for trend		<0.001		<0.001		0.018		0.013
Live microbes groups (*n* = 1,457)								
Low	Reference		Reference		Reference		Reference	
Medium	0.63(0.40, 0.99)	0.043	0.64(0.41, 1.02)	0.058	0.82(0.53, 1.26)	0.358	0.84(0.55, 1.27)	0.404
High	0.36(0.23, 0.57)	<0.001	0.37(0.23, 0.59)	<0.001	0.55(0.32, 0.95)	0.031	0.56(0.33, 0.94)	0.029
*P* for trend		<0.001		<0.001		0.025		0.022

*Crude model*: Not adjusted.

*Model 1*: Adjusted for age, sex, race.

*Model 2*: Adjusted for age, sex, race, marital status, education level, smoking status, drinking status, physical activity time (minutes), PIR, BMI.

*Model 3*: Adjusted for age, race, marital status, education level, smoking status, drinking status, physical activity time, PIR, BMI, CVD, Hypertension, DM, total energy, total protein and total fat.

COPD, chronic obstructive pulmonary disease; OR, odd ratio; CI, confidence interval; PIR, poverty income ratio; BMI, body mass index; CVD, cardiovascular disease; DM, diabetes mellitus.

## 4 Discussion

In this cross-sectional study, we investigated the association between dietary intake of live microbes and the prevalence of depression among patients with COPD. The results showed that compared to low intake levels, moderate to high dietary intake of live microbes was inversely associated with depression in COPD patients, with stable findings in subgroup and sensitivity analyses.

Previous studies have shown a significant association between the intake of live dietary microbes and mental health. For example, Chen et al. (19) found that high intake of live microbes was associated with lower rates of depressive symptoms among American adults. A randomized clinical trial revealed that probiotics substantially diminished anxiety and depressive symptoms when combined with antidepressants (36). Jin et al. (17) further indicated that the intake of live dietary microbes is linked to depressive symptoms and all-cause mortality in cancer survivors, suggesting potential benefits for mental health and disease prognosis. Additionally, Li et al. (18) discovered that live dietary microbes have a regulatory effect on depressive symptoms in patients with chronic inflammatory airway diseases (CIAD). These findings support the current study’s discovery that live dietary microbes may have a protective effect against depression. However, this study focuses specifically on COPD patients, further revealing the potential protective role of live dietary microbes in reducing depression rates among COPD patients. It is noteworthy that previous studies have found an L-shaped relationship between dietary probiotic intake and depression (27), may suggesting that there is an ‘optimal range’ for live dietary microbes in protecting against depressive symptoms. In contrast, our study found that as the amount of live dietary microbes consumed increased, the prevalence of depression continued to decrease. This linear relationship may be related to the unique pathophysiological characteristics of COPD patients. COPD patients often suffer from chronic inflammation and immune dysfunction, which may affect the regulatory effect of probiotic intake on depressive symptoms. Although we observed a linear trend, however, with only three intake categories and no *a-priori* threshold testing, we cannot exclude a non-linear shape. Future randomized controlled trials in COPD populations are needed to evaluate whether increasing dietary live microbe intake can reduce depression risk.

To verify the stability of the results, this study also conducted subgroup analysis and sensitivity analysis, and the results remained stable. In the subgroup analysis, this study found that the association between the intake of live dietary microbes and the incidence of depression remained significant among individuals under the age of 65, males, non-obese individuals, and patients without CVD. Although the association was more pronounced in certain subgroups (e.g., non-obese individuals), the interaction test results were not statistically significant (all interaction *p* values > 0.05). It is possible that younger COPD patients may possess a stronger adaptability and resilience of the gut microbiota, making the impact of live dietary microbes on their mental health more pronounced. Given that obesity and cardiovascular diseases themselves have a complex relationship with depression (37, 38), they may influence the occurrence of depression through mechanisms such as inflammation and metabolic disorders. In COPD patients without obesity and cardiovascular diseases, the protective effect of live dietary microorganisms might be more easily observed. Additionally, dietary live microbe intake may correlate with overall diet quality (e.g., higher fruit, vegetable, and fiber intake), which itself is associated with lower depression risk. Although we adjusted for total energy, protein, and fat intake, we could not account for overall dietary patterns such as the Healthy Eating Index (HEI) or DASH score. Thus, the observed association may partly reflect residual confounding by diet quality.

Although the mechanisms underlying this association are not fully understood. There are several possible explanations, for example, regulating gut microbiota and anti-inflammatory effects may be its main mechanisms of action. Patients with COPD often experience a state of chronic inflammation. This inflammatory response not only affects pulmonary function but may also impact the central nervous system through multiple pathways, thereby increasing the risk of depression (7). The association between inflammatory response and depression has been confirmed in multiple studies. Elevated levels of inflammatory factors are closely correlated with the severity of depressive symptoms (39). Live dietary microorganisms can regulate the immune system, reduce the levels of inflammatory factors, alleviate inflammatory responses, thereby reducing the risk of depression (40). Furthermore, in patients with COPD, dysbiosis of the gut microbiota has been confirmed to be associated with disease progression and related complications (41, 42). By ingesting live probiotics, the composition of the gut microbiota can be improved, increasing the quantity and diversity of beneficial bacteria, thereby enhancing lung health through the gut-lung axis (43). This improvement in gut microbiota may have a positive impact on depressive symptoms in COPD patients through the regulation of the gut-brain axis.

This study preliminarily elucidated the negative association between dietary live microbial intake and depression in COPD patients, providing a novel perspective for health management in this population. Recent studies underscore the need for personalized, multimodal strategies in complex depression (44, 45). Recent evidence also highlights the potential role of pharmacological augmentation strategies in treatment-resistant psychiatric conditions (46). Together, these findings reinforce our conclusion that integrating nutritional and microbiome-based approaches with existing pharmacological frameworks may offer the most promising path to alleviate depression in COPD patients. However, our study has some limitations. First, NHANES is a cross-sectional study, thus only associations can be observed, and causality cannot be inferred. Second, there is information bias due to recall bias from self-reported dietary intake and COPD/chronic bronchitis/emphysema diagnosis. In addition, because PHQ9 items overlap with COPD-related somatic complaints, potentially misclassifying respiratory disease as depression. Although this is the standard method of NHANES, relying solely on a single 24-h dietary recall to assess habitual intake is not optimal. Third, the conclusions of this study apply only to Americans and cannot be generalized to other populations. Forth, despite numerous adjustments for confounding factors, it may still be influenced by unknown confounders, for example, NHANES does not provide detailed lung function data or COPD severity classifications. Further large-scale prospective studies are needed in the future to use diet quality scores (such as HEI, DASH) for more comprehensive adjustments to establish causal relationships and mechanistic associations.

## 5 Conclusion

Our research indicates an inverse association between higher dietary intake of live microbes and the prevalence of depression among COPD patients aged 40 and above in the United States. However, future prospective studies are needed to verify this association.

## Data Availability

Publicly available datasets were analyzed in this study. This data can be found at: https://wwwn.cdc.gov/nchs/nhanes/Default.aspx.
